# Metagenomic analyses of bacteria on human hairs: a qualitative assessment for applications in forensic science

**DOI:** 10.1186/s13323-014-0016-5

**Published:** 2014-12-16

**Authors:** Silvana R Tridico, Dáithí C Murray, Jayne Addison, Kenneth P Kirkbride, Michael Bunce

**Affiliations:** Veterinary and Life Sciences, Murdoch University, Perth, WA 6150 Australia; Trace and Environmental DNA laboratory, Department of Environment and Agriculture, Curtin University, Perth, WA 6845 Australia; School of Chemical and Physical Sciences, Flinders University, GPO Box 2100, Adelaide, South Australia 5001 Australia

**Keywords:** Forensic, Metagenomics, Bacteria, Scalp hairs, Pubic hairs, Sexual assaults, Next-generation sequencing, 16S DNA

## Abstract

**Background:**

Mammalian hairs are one of the most ubiquitous types of trace evidence collected in the course of forensic investigations. However, hairs that are naturally shed or that lack roots are problematic substrates for DNA profiling; these hair types often contain insufficient nuclear DNA to yield short tandem repeat (STR) profiles. Whilst there have been a number of initial investigations evaluating the value of metagenomics analyses for forensic applications (e.g. examination of computer keyboards), there have been no metagenomic evaluations of human hairs—a substrate commonly encountered during forensic practice. This present study attempts to address this forensic capability gap, by conducting a qualitative assessment into the applicability of metagenomic analyses of human scalp and pubic hair.

**Results:**

Forty-two DNA extracts obtained from human scalp and pubic hairs generated a total of 79,766 reads, yielding 39,814 reads post control and abundance filtering. The results revealed the presence of unique combinations of microbial taxa that can enable discrimination between individuals and signature taxa indigenous to female pubic hairs. Microbial data from a single co-habiting couple added an extra dimension to the study by suggesting that metagenomic analyses might be of evidentiary value in sexual assault cases when other associative evidence is not present.

**Conclusions:**

Of all the data generated in this study, the next-generation sequencing (NGS) data generated from pubic hair held the most potential for forensic applications. Metagenomic analyses of human hairs may provide independent data to augment other forensic results and possibly provide association between victims of sexual assault and offender when other associative evidence is absent. Based on results garnered in the present study, we believe that with further development, bacterial profiling of hair will become a valuable addition to the forensic toolkit.

## Background

Over the last decade, the development of bacterial culture-independent approaches (metagenomics), based on 16S rRNA genes (hereafter referred to as 16S), sequences has become the cornerstone of microbial ecology [1]. The advent of next-generation sequencing (NGS) technologies and platforms capable of generating millions of sequences per sample facilitated assessments of microbial communities between body sites and individuals [2,3]. The increased sequencing power stimulated the development of robust computational programmes capable of processing large, complex sequencing data sets [4] and enabled phylogenetic analyses of human and environmental genomes [5,6].

Studies on the human microbiome (the collective genomes present in the human body) suggest that there are significant differences in bacterial composition not only between different body sites but also between individuals [3,5,7]. The potential that individuals may harbour unique bacterial species is of significance to forensic investigations.

For centuries, associative hair evidence relied solely on comparative microscopy based on qualitative features such as colour and pigmentation [8-10]. The advent of PCR in the mid-1980s initiated a paradigm shift in the forensic examination of hairs. For the first time, DNA profiles could complement qualitative microscopical observations [11]. However, the success of the highly discriminatory short tandem repeat (STR) profiling is dependent on hairs bearing anagen roots (actively growing hairs) that are rich in nuclear DNA (nuDNA) and, to a lesser extent, hairs that are in the quiescent (catagen) growth phase [12]. However, the majority of hairs recovered in forensic investigations are shed hairs (i.e. those in their telogen phase); these hairs have ceased to grow and contain little or no nuDNA [13]. STR profiling of these hair roots typically yields trace amounts of, often degraded, human DNA and can require the use of low-template DNA strategies and the complications that accompany such approaches [14]. In these instances, mitochondrial DNA (mtDNA) analysis is routinely conducted. However, due to its common matrilineal inheritance and haploid nature, mtDNA typing yields modest exclusionary capability, which lacks the statistical power afforded by STR profiling [15]. However, low yields of human nuDNA from forensic hair samples does not equate to the absence of other sources of DNA that could assist in the individualisation of hair. Indeed, metagenomic analyses of hairs unsuitable for nuDNA profiling may provide a microbial fingerprint to augment other forensic results such as mtDNA analyses. This would not involve extra or additional extraction procedures, as DNA isolation procedures for human DNA will also ‘collect’ microbial DNA.

Conventional forensic hair examination, using either morphological or molecular techniques, is contingent upon the deposition and recovery of hairs; however, despite Locard’s adage that ‘every contact leaves a trace’ [16], this may not always be the case. Research in relation to the transfer of pubic hairs in forensic investigations involving sexual assault cases discovered limited transfer (4%) of male pubic hair to female genital area during sexual intercourse (SI) [17]. In addition, the present study demonstrated that no female pubic hair transfer to male genital area took place.

The utility of metagenomic analyses for forensic applications has been explored since the inception of NGS; for example, Fierer et al. [18] conducted preliminary work to explore the potential to link individuals to computer keyboards and mice on the basis of transfer of skin bacteria. However, one of the most ubiquitous of evidence types—human hair—has yet to be evaluated in the context of forensic metagenomics. To the best of our knowledge, this present study is the first to qualitatively assess the viability of metagenomic analyses of hairs in a forensic context. The three aims of the research reported here were to assess:

1. Whether human scalp and pubic hairs can be differentiated on the basis of their 16S microbial composition
2. Whether individuals can be differentiated on the basis of microbial taxa colonising scalp and pubic hairs
3. Whether bacterial 16S profiles on hair shafts are stable over time

Overall, the objective of this initial study was to establish whether further development of the technique is warranted.

## Methods

### Sample collection

Bacterial communities, associated with human scalp and pubic hair, were surveyed using a multiplex barcoded sequencing approach from seven healthy Caucasian individuals of both sexes (two of whom were in a *de facto* relationship), ranging in age from 23 to 53 years old. The health status of each volunteer was self-reported with each individual stating that antibiotics were not taken at least 8 months prior to the collection of hairs used in the study. Each individual self-collected a number of hairs cut from the scalp and pubic areas, at three time points, initial collection in addition to 2 and 5 months thereafter, referred to as T0, T2 and T5 respectively. Replication is important in NGS amplicon sequencing workflows—due to the investigative nature of this study and limited availability of resources, we selected to investigate multiple time points (temporal replicates) in lieu of multiple extractions at each time points (sampling replicates).

Each volunteer was provided with a hair collection kit consisting of labelled clip-seal plastic bags, sterilised scissors, ethanol wipe, latex disposable gloves and disposable forceps. Hairs were cut close to the skin and from approximately the same area at each time point; volunteers were asked to clean the scissors with ethanol wipes between sampling to avoid contamination. The rationale of severing the hairs close to the skin, rather than plucking the hairs was to ensure the bacterial taxa identified were more likely to originate from the hair shaft rather than the skin. Hairs taken from the head were labelled as female scalp hair (FSH) or male scalp hair (MSH); similarly, female and male pubic hairs were marked female pubic hair (FPH) or male pubic hair (MPH). Once sampled, these hairs were placed in separate labelled clip-seal plastic bags and stored at room temperature after being catalogued. Hairs were sampled and processed within 24 h of collection, and unused hair samples were returned to their original packaging and stored at room temperature; these hairs were not further sampled or examined. The effect of storage and storage conditions on bacteria was not in the scope of this present study; however Lauber et al. [19] investigated the effects of storage conditions on bacteria and concluded that bacterial community composition is unaffected in the short-term.

Each volunteer was made aware of the nature of the study and gave written, informed consent. Information regarding the sexual habits or orientations of the volunteers was not sought. The project was approved by, and conducted in accordance with, Murdoch University Human Research Ethics Committee Policies and Guidelines (Project Number 2011/139).

### DNA extraction and quantification

Three hairs from each body area were cut into approximately 1 cm lengths and placed into 1.5 ml Eppendorf tubes. The contents of each tube were digested overnight using 1 ml of hair digest buffer containing: 10 mM Tris pH 8 (Sigma, St. Louis, Mo, USA), 10 mM NaCl (Sigma), 5 mM CaCl_2_, (Sigma), 2.5 mM EDTA pH 8 (Invitrogen, Carlsbad, CA, USA), 1 mg/ml ProK (Amresco, Solon, OH, USA), 40 mM DTT (Thermo Fisher Scientific, Waltham, MA, USA) 2% SDS (Invitrogen) and milliQ water (Sigma) to make the remaining volume. The samples, including extraction and laboratory environmental controls, were secured on rotating arms (to ensure total immersion) and digested overnight in a 55°C oven.

All samples were then centrifuged for 2 min at 13,000 rpm. To concentrate the DNA, a total of 600 μl of supernatant was transferred to Vivaspin ultrafiltration spin columns with a 30,000 MW cutoff (Sartorius Stedim Biotech, Göttingen, Germany) and centrifuged at 30,000 rpm to leave 50–100 μl of supernatant. Concentrated supernatant was subsequently combined with five volumes of PB buffer (Qiagen, Valencia, CA, USA) and transferred to a Qiagen silica spin column and centrifuged at 13,000 rpm for 1 min. Two wash steps followed (Qiagen AWI buffer and AWII buffer) prior to elution of DNA from the spin column with 60 μl of 10 mM Tris-Cl pH 8 buffer. The DNA extracts were subsequently quantified via real-time quantitative polymerase chain reaction (qPCR; Applied Biosystems StepOne, Foster City, CA, USA) using SYBR green and the bacterial 16S F515/Bact 16S (V4 loop)_R806 primers (Table 1).

**Table 1:** **Details of primers used in the amplification of 16S RNA region of bacterial mitochondrial genome**

	**Primer sequence (5′ to 3′)**	**Reference**
Forward bacterial primer (Bact_16S_F515)	GTGCCAGCMGCCGCGGTAA	Turner et al. [20]
Reverse bacterial primer (Bact_16S_R806)	GGACTACHVGGGTWTCTAAT	Caporaso et al. [21]

Extracts were analysed using qPCR for neat extracts in addition to 1/10 and 1/100 dilutions, in order to determine if extractions were successful and to identify samples with low-template DNA (defined as those with CT values >32). The possible presence of PCR inhibitors was also determined by qPCR. The 16S qPCR assay was conducted in 25 μl reactions using a 2X ABI Power SYBR master mix (Applied Biosystems) together with 2 μl of extracted DNA with primer concentration at 0.4 μM (IDT) cycled for 95°C for 5 min followed by 50 cycles of 95°C for 30 s, 55°C for 30 s, 72°C for 45 s, with 1°C melt step and a 10-min final extension at 72°C. The optimal DNA concentration free of inhibition was used for all subsequent analyses. Each hair sample bacterial extract had CT values less than 32 PCR cycles indicating the presence of sufficient 16S template copy number for robust NGS amplicon sequencing.

### Fusion-tagged 16S V4 Amplicon generation

Bacterial F515 and R806 (Table 1) 16S primers (targeting the V4 region) used in the initial qPCR extract screen, giving a size variable product minus primers of ~250 base pairs, were modified into fusion primers for the generation of amplicon products for subsequent sequencing. Each fusion primer consisted of a GS FLX Titanium (Lib-A) adapter A or B on the 5′ end followed by a unique 6 bp multiplex identifier (MID) tag and the template specific forward or reverse primer at the 3′ end of the primer [22] giving a final size variable product of ~350 bp including primers and additions. A single-step, uniquely tagged fusion PCR approach was employed to minimise the contamination associated with the multiple PCR steps used in NGS workflows [20].

For each time point, each extract was assigned a unique 6 bp MID-tagged fusion primer in preparation for amplicon sequencing. MID-tagged amplicons were generated in triplicate (i.e. PCR replicates) in 25 μL reactions containing: 1X PCR Gold Buffer (Applied Biosystems), 2.5 mM MgCl_2_ (Applied Biosystems), 0.1 mg/ml BSA (Fisher Biotech, WA, Australia), 0.25 mM of each dNTP (Astral Scientific, NSW, Australia), 0.4 μM of forward and reverse primer (IDT), 0.2 μL1 unit of Taq DNA polymerase (AmpliTaq Gold™), 1:80,000 (final concentration) of SYBR Green ‘gel-stain’ (Life Technologies, S7563, Carlsbad, CA, USA) and DNA extract. The same processes were performed on PCR negative/reagent controls for each PCR plate run, pre- and post addition of DNA extracts.

All PCR amplicons were purified using the Agencourt AMPure XP™ Bead PCR Purification protocol (Beckman Coulter Genomics, Danvers, MA, USA). Solely for the purpose of sequencing coverage, purified amplicons were electrophoresed on 2% agarose gel to obtain roughly equimolar ratios of each sample. Where extraction/environmental controls or PCR negative/reagent controls showed positive qPCR results, these were also pooled, purified and sequenced. The final pooled library was quantified using qPCR to determine the appropriate volume of library to use for emulsion PCR (emPCR) prior to amplicon sequencing on the GS Junior™ (described in Murray et al. [21], using reaction conditions in Murray et al. [23]). All emPCR, bead recovery and amplicon sequencing procedures were carried out according to Roche GS Junior™ protocols for amplicon sequencing (Lib A).

### Bioinformatic analysis

Amplicon sequence reads obtained from the GS-Junior™ (hereafter referred to as sequences) were sorted into batches based on MID tags assigned to each extract allowing for no mismatch in MID tag DNA sequences. Additionally, template-specific 16S bacterial primer sequences were annotated and trimmed from all sequences allowing for no mismatch in base composition or primer sequence length. Sequences that failed to meet these criteria were discarded. The aforementioned steps were conducted using Geneious™ v7.0.6 [24].

Once batched and trimmed, sequence FASTA files were imported into QIIME V1.8.0 [25] and merged into a single FASTA file. Chimeric sequences were identified and removed on a per individual sample basis using the usearch61 [26] *de novo* method passing --split_by_sampleid. Following this, operational taxonomic units (OTUs) were identified using an open reference OTU picking method using usearch61 with a 97% clustering identity, using the most abundant sequence within each OTU as the representative sequence and the Greengenes 13.8 database release [27]. Representative sequences for each OTU were aligned using PyNAST [28] against the Greengenes 13.8 pre-aligned database, the alignment filtered and phylogeny built using FastTree [29] in QIIME. Additionally, any OTUs found within the control samples of specific time points (i.e. T0, T2, T5) were removed from samples contained within the respective time point. Following the removal of control OTUs, each individual sample was filtered to remove low abundant OTU clusters. In each case, all singleton OTUs were discarded and any OTU whose abundance was below 0.2%, an estimated error rate associated with 454 sequencing [30], of the total number of filtered sequences in that sample was removed. OTUs remaining post-filtering were taxonomically identified using the BLASTn option within QIIME’s assign taxonomy script against the Greengenes 13.8 database. Moreover, OTUs were taxonomically assigned using RDP [31] and UCLUST [26] options, again against the Greengenes 13.8 database [27].

To determine at a gross level if there was clustering of samples according to sex and/or somatic origin, a principal co-ordinate analysis (PCoA) plot was constructed using filtered sequences negating whether or not sequences were part of the core microbiome.

Following taxonomic identification and PCoA construction, the core microbiome for each sex/somatic origin (SSO) grouping (i.e. FSH, FPH, MSH and MPH) was determined using QIIME. The ‘core’ microbiome was defined in accordance with that established by Shade et al*.* [32], all OTUs that occur in two or more (i.e. the majority) of the recorded time points for each of the SSO groupings. Any OTU’s occurring in only one of three time points is classed as ‘transient’ (Tr). In addition to this, the number of OTUs that were unique to an individual was determined; these were defined as all OTUs occurring solely in that individual across at least two time points irrespective of whether it was found to be a core OTU in the above SSO groupings. Upon identification of personalised OTUs, it was determined whether or not the said OTUs occurred in pubic hair, scalp hair or in both. Finally, the number of OTUs shared solely by two individuals was identified to examine whether the number of OTUs between the cohabiting couple was greater in comparison to other non-co-habiting participants.

## Results and discussion

Forty-two pools of DNA extracts obtained from human scalp and pubic hairs were used to interrogate their microbial composition by next-generation sequencing. A total of 79,766 reads were generated, yielding 39,814 reads post control and abundance filtering. On average, the coverage per sample was 1,899 reads pre-filtered and 948 post-filtered. Whilst this depth of coverage is less than ideal given the advancement in NGS technology (i.e. Illumina and Ion Torrent platforms), these 454 data are still sufficient to explore the potential of hair microbial forensics for future development. Like all novel forensic techniques, metagenomic analyses of hairs will ultimately require robust evaluation and validation to ensure that these analyses are fit for purpose and able to withstand scientific scrutiny. Part of this validation should take into consideration: replication (spatial, temporal and PCR replicates); persistence of hair bacteria not only once they are transferred or deposited (during contact and stability during storage) and prevention of contamination during processing hairs in the laboratory. Budowle et al. [33] outline and discuss in detail the future validation criteria for metagenomic analyses in relation to microbial forensic applications, which they believe will require international participation. However, such an undertaking is beyond the scope of this initial evaluation into just one of many applications of forensic metagenomic investigations.

There are many ways to present metagenomic data such as generated here; the sections below explore the data using PCoA, taxonomy and OTU’s focusing on the value of the data in forensic applications. OTUs taxonomically assigned using RDP or UCLUST options revealed little to no difference in assignment to the rank of family. For this reason, all assignments refer to BLAST taxonomic assignments.

### Principal coordinates plot

**Figure 1:**
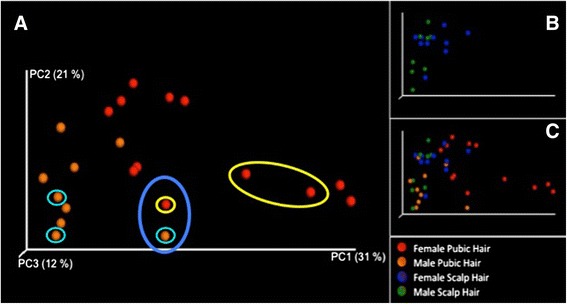
**Principal coordinate plots (PCoA).** Clustering of microbial taxa from each individual at each collection time point. The lilac circle represents post-SI bacterial sequences, whilst the pale blue and yellow circles represent non-SI bacterial sequences—both circles relate solely to the co-habiting couple. **Panel A** represents pubic hair microbial taxa from male (orange) and female (red) participants. **Panel B** represents scalp hair microbial taxa from male (green) and female (blue) participants. **Panel C** represents microbial taxa present in male and female scalp and pubic hair samples.

Of all the data generated in this study, the NGS data generated from pubic hair held the most potential for forensic applications. A general dichotomy was observed between taxa (OTUs) harboured on male and female pubic hair shafts (Figure 1).

In general, males were clustered close to the PC2 axis along the PC1 axis whilst females were more evenly spread along the PC1 axis and further from the PC2 axis than the males. Data relating to two individuals, who were a cohabiting couple, presented some interesting results. The red dots in the yellow ellipse at high PC1 represents the taxa present on the female partner of the couple at T0 and T2 whilst the two orange dots enclosed by small blue circles at low PC1 represent the taxa from the male partner at T0 and T2. The lilac circle encloses one red dot (taxa from the female at T5) and one orange dot (taxa from the male at T5). Microbial taxa extracted from the male and female at this time point were more similar to each other than to their other previous time points (T0, T2). Discreet enquiries revealed, unlike the preceding time points; the couple in question had engaged in sexual intercourse prior to the collection of T5 hair samples. It is noteworthy that intercourse had taken place 18 h prior to the collection of pubic hairs and both individuals had showered in the interim period. Cross-transference of bacteria during intercourse may account for the variation in taxa observed. Cross-transference, or shedding of skin micro flora, is not uncommon for individuals sharing living or communal spaces [34] or during contact sports in which Meadow et al. [35] observe ‘Our results are consistent with the hypothesis that the human skin microbiome shifts in composition during activities involving human to human contact’. The results we present here suggest that the pubic hair microbiome might be quite stable, even during cohabitation, but it might be shifted dramatically during sexual intercourse for some time. This present study is the first to suggest cross-transference of pubic/genital microbial taxa as a result of intercourse. Although further analyses need to be conducted, this initial finding bodes well for future forensic applications involving sexual crimes.

An additional advantage is that compared to other body areas such as the skin, gastrointestinal tract (GIT) and mouth, fewer bacterial species seem to comprise the vaginal microbiome [36]. The advantage of simpler communities and fewer taxa in the vaginal microbiome is one that may facilitate forensic investigations by providing results in a timely manner.

The clear microbial distinctions between pubic hairs from the sexes may largely be attributable to the prevalence of *Lactobacillus* spp. in the female pubic hair samples and the absence of these bacteria in the male samples (excepting the co-habiting male at T5) (Figure 1). Additionally, male pubic hair microbial taxa were clustered along axis PC2 suggesting that these taxa (OTUs) were common to the male microbiota.

In contrast, female pubic hair bacterial taxa showed elongation along axes PC1 and PC2. The elongation of data along PC1 may be attributable to females harbouring different lactobacilli species (Tables 2 and 3). However, the concomitant elongation of data along axis PC2 suggests the presence of secondary differences, differences that may be due to the presence of personalised taxa (Table 3).

**Table 2:** **Shared taxa from pairwise comparisons of all data located in scalp (Sc) and/or pubic (Pu) hairs**

**Individual**	**1 (female)**	**2 (male)**	**3 (male)**	**4 (female)**	**5 (female)**	**6 (female)**	**7 (male)**
1 (Female)		*Xanthomonadaceae* (Sc)	*Dialister* spp. (Pu)	*Lactobacillus iners* (Pu)	0	0	0
				*Prevotella* spp. (Pu)			
				*Peptinophilus* spp. (Sc/Pu)			
2 (male)			0	0	***Betaproteobacteria*** **(Sc/Pu)**	*Actinomycetales* (Pu), *Neisseriaceae* (Sc)	*Dietziac* (Sc)
					***Neisseriaceae*** **(Sc)**	*Mycoplana* spp. (Sc/Pu)	*Knoellia subterranea* (Sc/Pu)
					***Corynebacteriaceae*** **(Pu)**		
					***Lactobacillus*** **spp. (Pu) X4**		
3 (male)				*Bifidobacterium* (Pu/Sc)	0	0	*Corynebacteriaceae* (Sc)
				*Anaerococcus* (Pu)			*Paracoccus* (Sc/Pu)
4 (female)					*Lactobacillus* spp. *X*2 (Pu)	*Limnohabitans* spp. (Sc)	0
					*Rhodobacteriaceae* (Sc)		
5 (female)						0	0
6 (female)							*Corynebacterium* spp. (Sc/Pu)
							*Aggregibacter segnis* (Pu)
7 (male)							

The cohabiting couple (bold) share more taxa, including multiple lactobacilli species, than other individuals.

**Table 3:** **Personalised (unique) bacterial taxa colonising male and female scalp and pubic hair**

**Somatic origin (sex/individual)**	**Bacterial taxa**	**Natural habitat**
Pubic (F. Ind.1)	*Lactobacillus* spp. ×1^a^ (*Lactobacillaceae*)	Most prevalent genera in human vagina; also present in gastro-intestinal tract (GIT) [39]
Scalp (F. Ind.1)	*Neisseriaceae*	Normal human flora of oro-nasopharynx [39]
Scalp (M. Ind.2)	*Nocardioidaceae*	Soil and aquatic habitats [44]
Scalp (M. Ind.2)	*Streptococcus sobrinus* (*Streptococcaceae*)	Implicated in dental pathologies [39]
Pubic (M. Ind.3)	*Corynebacterium* × 2 spp.^a^ (*Corynebacteriaceae*)	Major inhabitants of skin flora [39]
Pubic (M. Ind.3) spp.	*Tissierellaceae fam.nov.*	Intestine, vagina, oral cavity; some general environmental [46]
Pubic (M. Ind.3)	*Anaerococcus* spp.^a^ (*Tissierellaceae fam.nov*)	Human nasal cavity, skin and vagina [47]
Pubic (F. Ind.4)	*Campylobacteraceae*	Human oral cavity, intestinal tracts and environmental [48]
Scalp (F. Ind.5)	*Rhodocylcaceae*	Activated sludge and waste water [49]
Scalp (F. Ind.5)	*Micrococcaceae*	Widely distributed in environment; commensal on human skin [39]
Pubic (F. Ind.5)	*Lactobacillus* × 3 spp.^a^ (*Lactobacillaceae*)	Prevalent and abundant in human vagina; also present in GIT [39]
Pubic (F. Ind.6)	*Gardnerella* spp.^a^ (*Bifidobacteriaceae*)	Implicated in bacterial vaginosis (BV) [39]
Pubic (F. Ind.6)	*Enterobacteriaceae*	Many species harmless symbionts of the human GIT [39]
Pubic (F. Ind.6)	*Methylobacteriaceae*	Ubiquitous in nature, opportunistic pathogens [50]
Pubic (F. Ind.6)	*Pasteurellaceae*	Respiratory, alimentary and reproductive tracts [51]
Pubic (F. Ind.6)	*Pseudomonas* spp.^a^ (*Pseudomonadaceae*)	Transients on human skin [39]
Pubic (M. Ind.7)	*Aurantimonadaceae*	Marine environment [52]
Scalp (M. Ind.7)	*Brachybacterium* spp.^a^ (*Dermabacteraceae*)	Variety of environments [53]
Scalp/Pubic (M. Ind.7)	*Gordoniaceae*	Majority environmental [54], some human pathogens [55]
Scalp/Pubic (M. Ind.7)	*Rhodobacteriaceae*	Aquatic habitats [56]
Scalp/Pubic (M. Ind.7)	*Sphingomonadaceae*	Widespread in nature; present in water [57, 58]

Data shows sex and somatic origin of hairs that harboured personalised bacterial taxa, as well as the natural habitats of the taxa.

^a^Bacteria identified to genus or species are regarded as the most discriminatory. As such, these taxa may be of significance in forensic investigations.

The PCoA plot of male and female scalp hair microbiota over the 5-month time period did not demonstrate any significant clustering (Figure 1). This is most likely attributable to male and female scalp hairs harbouring similar bacterial taxa. However, some of the female taxa are slightly spread out along axis PC1 suggesting that there may be some variation in microbial taxa in the hairs of these individuals. The distribution and composition of the microbial communities colonising scalp and pubic hair is discussed in further detail below.

### Hair microbiota

**Figure 2:**
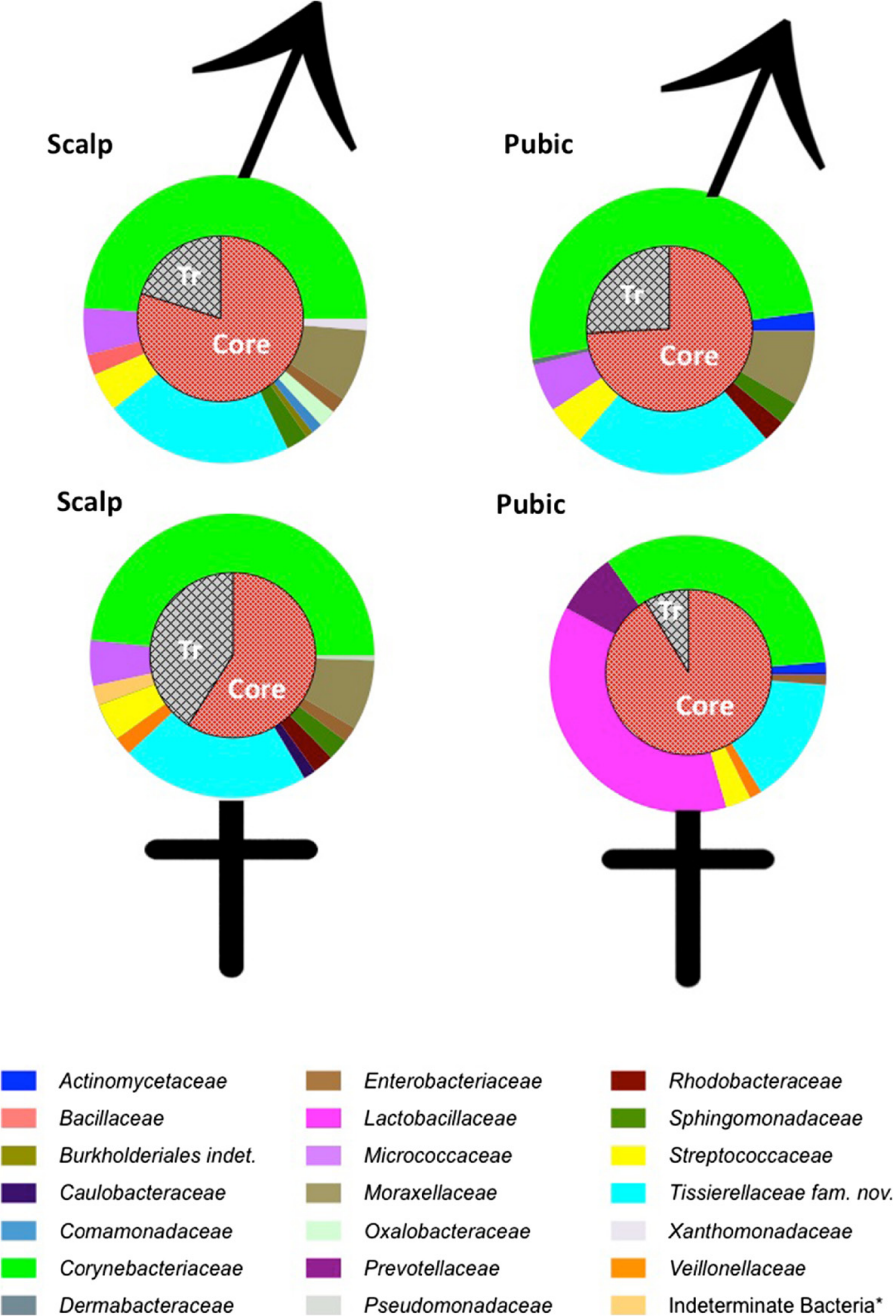
**Microbial data extracted from scalp and pubic hairs.** Diagrams illustrating core and transient (Tr) bacterial taxa on male and female scalp and pubic hair samples.

Bacteria colonising male and female scalp and pubic hair samples are classed as either ‘core’ or transient (Tr) bacteria (Figure 2, see the ‘Methods’ section). In relation to the number of OTUs extracted from scalp and pubic hair microbiomes, far less bacterial sequences were lost post control filtering for pubic microbiomes in comparison to scalp hair. Pubic hairs in general contained more OTUs than scalp hair (approximately 50 male OTUs/55 female for scalp hairs c.f. approximately 73/76 for pubic hairs). Therefore, in general, pubic hair microbiomes appear to be less influenced by environmental bacteria than scalp hairs and possible harbour more niche specific bacteria. Zhou et al. [37] support this premise by demonstrating that (in comparison to other areas of the body) vaginal microbiota consisted of less stable bacteria (i.e. more transient bacteria) and showed lower alpha diversity (i.e. low species richness), supporting the premise of pubic hair harbouring niche specific bacteria.

### Pubic hair microbiota

Male pubic hairs could be readily distinguished from female pubic hairs on the basis of their respective microbiota. *Lactobacillus* was the most prevalent taxon that clearly differentiated male and female pubic hair microbiota (Figure 2). Whilst the prevalence of *Lactobacillus* spp. in the vagina and vaginal secretions is well established [38-40], this present study is the first to discuss these bacteria colonising pubic hairs, and general pubic area, in the context of probative value in forensic investigations. Fleming and Harbison [41] suggested the presence of two *Lactobacillus* spp. (*Lactobacillus crispatus* and *Lactobacillus gasseri*) as suitable forensic markers to identify vaginal secretions. However, microbial data garnered in this present study suggest that a NGS metagenomic approach may be preferable to those that target specific species. The variety of *Lactobacillus* spp. detected in pubic hairs from the female cohort consisted of 11 OTUs (taxa) in total; three *Lactobacillus* spp. were unique to Female 5, one *Lactobacillus* spp. occurred in Female 1, and four *Lactobacillus* spp. were uniquely between the cohabiting couple. In addition, two *Lactobacillus* spp. were uniquely shared between F4 and F5, and one OTU was uniquely shared between F4 and F1 (Tables 2 and 3).

Compared to male pubic hairs, female pubic hairs harboured fewer transient bacteria (Figure 2); the number of bacterial sequences comprising transient bacteria of female pubic hairs was approximately half the number of those found in male pubic hair (Table 4). This disparity may be attributable to lactobacilli conferring ‘antimicrobial protection’ to the vagina by preventing colonisation by other microorganisms [38]. Li et al. [42] also found that in comparison to other body areas, the vaginal microbiome is less transient (i.e. more stable). This stability was apparent in the differences between the number of OTUs detected in the scalp and pubic hair controls; there were significantly less OTUs present in controls from the pubic hairs in comparison to the scalp hairs. Post control filtering for FSH and MSH samples there were 33% and 43% (respectively) of sequences left. In comparison, for FPH and MPH there were 70% and 72% (respectively) sequences left, post-filtering. The disparity between the two somatic origins suggests that the bacterial taxa in scalp hair extracts had a high proportion of environmental bacteria that readily appear in controls.

**Table 4:** **Number of bacterial 16S sequences found in core microbiomes compared with transient number of sequences for each sex/somatic origin sampled**

**Sex/somatic origin**	**Core bacteria**	**Transient bacteria**	**Total bacteria** ^**a**^
Female scalp hair	3,123	2,162	5,285
Female pubic hair	16,019	1,524	17,543
Male scalp hair	4,838	1,220	6,058
Male pubic hair	8,109	2,819	10,928

Female pubic hair harboured less transient bacteria, but more core bacteria than male pubic hair.

^a^Total number of sequences found to remain once sequences found in controls and low abundant clusters were removed.

### Scalp hair microbiota

In contrast to the pubic hairs, scalp hair microbiota showed no correlation with the sex of the donor (Figure 2). Male and female scalp hair bacterial taxa consisted of normal human skin commensals, e.g. *Anaerococcus* spp., and environmentally derived taxa, e.g. Knoellia subterranea, many of which occurred in both male and female samples (Table 5). In the present study, the most significant difference observed in male and female scalp hairs was the disparate proportions of the transient bacterial taxa (Figure 2). Almost twice as many transient bacterial taxa were present in female scalp hair compared to males (Table 4). This may be due to the greater frequency of females grooming and/or washing and/or dyeing or bleaching their hair in comparison to males. Such grooming practices may prevent establishment of more stable bacterial colonies in favour of less stable (transient) bacterial colonies. Irrespective of the cause of this disparity, this observation cannot be regarded significant in relation to forensic investigations.

**Table 5:** **Natural habitats of shared bacterial taxa identified by pairwise comparisons**

**Shared bacterial taxa**	**Habitat**
*Aggregibacter segnis* (*Pasteurellaceae*)	Normal human oral flora [51]
*Anaerococcus* spp. (*Tissierella nov.fam.*)	Commensal human flora, opportunistic pathogens also environmental [46,47]
*Betaproteobacteria*	High order taxon (Class). Members largely environmental, also include human pathogens and commensals [60]
*Bifidobacterium* spp. (*Bifidobacteriaceae*)	Normal intestinal flora [39]
*Corynebacterium* × 1 spp. (*Corynebacteriaceae)*	Key members associated as part of skin flora [39]
*Dialister* spp. (*Veillonaceae*)	Implicated in oral cavity diseases [39]
*Dietziaceae*	Environmental and implications as an emerging human pathogen [48]
*Knoellia subterranea* (*Intrasporangiaceae*)	Environmental [61]
*Lactobacillus iners* and *Lactobacillus* spp. ×6 *(Lactobacillaceae*)	Part of a suite of lactobacilli that inhabit human female genital-urinary area [40]
*Mycoplana* spp. (*Caulobacteraceae*)	Environmental [62]
*Neisseriaceae*	Members may be human commensals (of the mouth) or pathogenic [39]
*Paracoccus* (*Rhodobacteriaceae)*	Environmental [49]
*Peptinophilus* spp. (*Tissierella fam.nov*.)	Pathogenic [47]
*Prevotella* spp. *(Prevotellaceae)*	Oral, vaginal and GIT commensals [39]
*Xanthomonadaceae*	Plant pathogen (opportunistic human pathogen) [59]

The taxa and habitats relate to the data provided in Table 2.

Costello et al*.* [3] identified two dominant 16S sequences from scalp swabs: *Propionibacterinae* in which members are predominant bacteria in hair follicles and other sebaceous sites [39] and *Streptophyta* (a plant phylum). In contrast, the predominant bacterial taxa from hair shafts in this study were *Corynebacteriaceae* and *Tissierellacea fam.nov* (‘new family’) (Figure 2). The difference may be attributable to either environmental differences (i.e. different study sites) or the collection technique employed by Costello et al*.* [3] where swabbing the top of the head might have favoured the removal of scalp/follicular bacteria (i.e. propionibacteria rather than hair shaft bacteria).

### Personalised and shared bacterial taxa

Forensic investigations seek to establish ‘common origin’ or ‘source attribution’ of evidence, that is, to establish with reasonable scientific certainty that a particular individual is the source of an evidentiary sample. In relation to biological evidence, this question may be addressed through the detection of individualising biological characteristics, for example, a human DNA profile, characteristics which excludes other individuals as being the source. Ideally, these characteristics should not commonly occur within the general population or one that is solely found in males or females.

**Figure 3:**
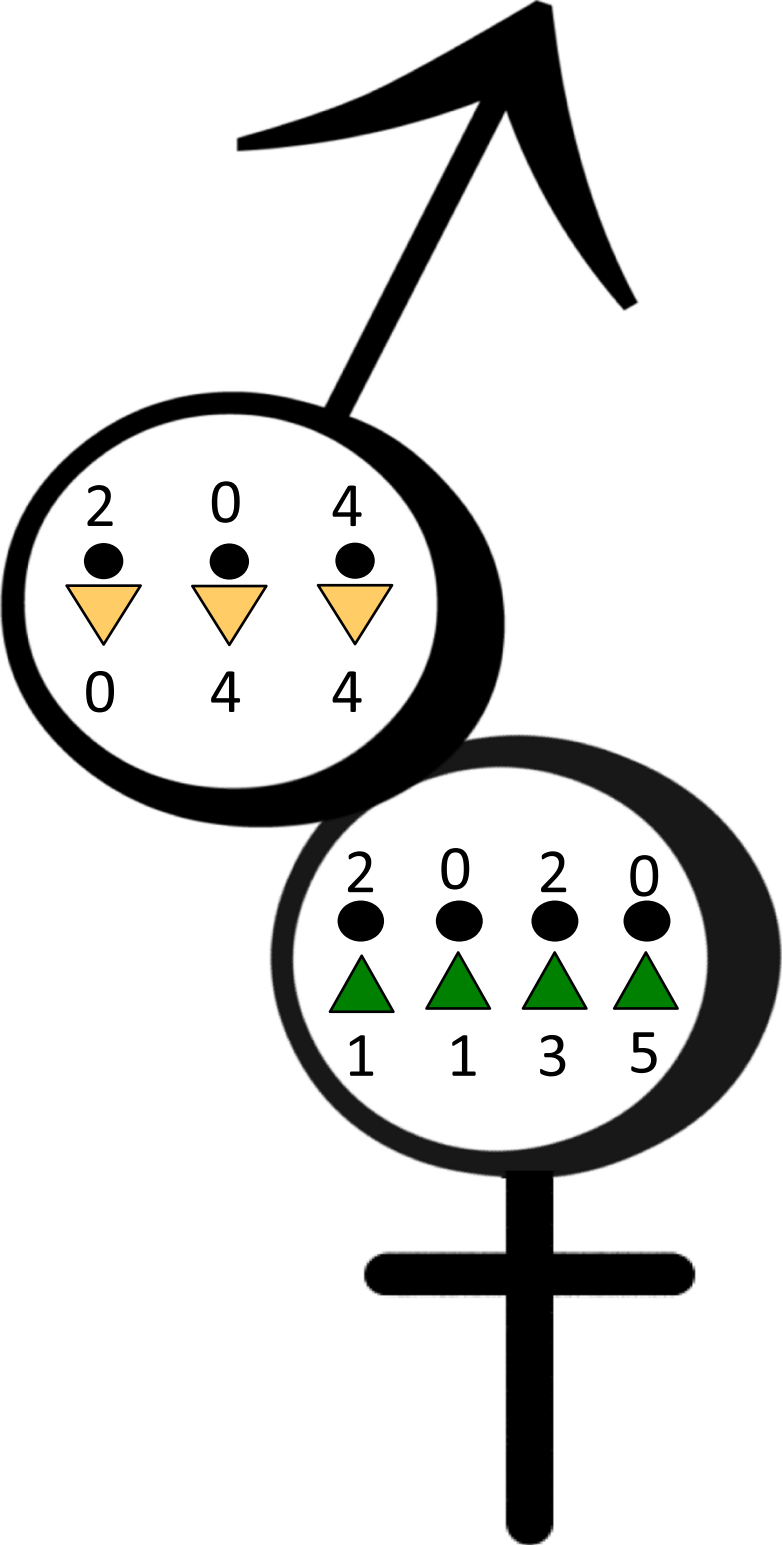
**Personalised microbial data.** Diagrammatic summary of unique bacterial taxa found in male and female scalp and pubic hair samples. Male individuals left to right: Individuals M2 (cohabiting male), M3 and M7. Female individuals left to right: Individuals F1, F4, F5 (cohabiting female) and F6.

Inside the confines of the 16S V4 region, with the exception of one male (co-habiting male at T5), all individuals harboured unique taxa on their pubic hairs (Figure 3). In addition to personalised bacteria that were part of the normal skin flora, e.g. Corynebacteriaceae, pubic hairs were also colonised by environmentally derived bacterial taxa, e.g. Methylobacteriaceae (Table 3).

Hairs from scalp and pubic regions, for both sexes, included shared taxa that are common inhabitants of human skin or scalp, e.g. Corynebacteria, or were environmental in origin, e.g. Rhodobacteriaceae (Tables 2 and 5). At first glance, the commonality of these bacteria may appear to be of minimal probative value; as discussed in a preceding section, personalised features should be uncommon traits or features. However, common bacteria may harbour single nucleotide polymorphisms (SNPs) within their genome, which may further discriminate between individuals**.**

Among all mammals, the microbiota composition is extensively conserved at the high taxonomic levels such as phylum or class. At these taxonomic levels, humans are very similar to each other (and other mammals) but variation increases progressively at the lower taxonomic levels. Personalised taxa, which allow discrimination between individuals (the goal of forensic applications), are likely to be detected at these lower taxonomic rankings. Personalised taxa may be present in high or low abundance; detection of low abundance taxa may only be detected by ultra-deep sequencing of the extracted bacterial DNA. In this regard, higher depth of coverage afforded by NGS platforms such as Ion Torrent or Illumina may be more informative than the 454 data presented here. As Ursell et al*.* [43] noted **‘**it is important to realise that sampling depth may be critical for distinguishing taxa that are absent from those that are merely rare’. Under these circumstances, it is critical to discount bacterial taxa present in all control samples in order for the results to be not only robust but also scientifically accurate and capable of withstanding scientific and legal scrutiny.

Temporal stability data garnered in this study broadly suggest that bacteria on scalp hairs may be more prone to fluctuations in comparison to pubic hairs (in addition to being more prone to environmental contaminants). The data shows that, on average and post-filtering, approximately 17% (range 6%–25%) of pubic hair bacterial OTUs were temporally stable across all time points; whilst, on average, scalp hair harbour approximately 5% (range 0%–13%) of bacterial OTUs (Table 6). These preliminary findings suggest that pubic hair bacteria may be more temporally stable than scalp hair bacteria and therefore potentially of more probative value than scalp hair bacteria.

**Table 6:** **Temporal stability of bacterial taxa (OTUs)**

	**FSH 1**	**FPH 1**	**MSH 2**	**MPH 2**	**MSH 3**	**MPH 3**	**FSH 4**	**FPH 4**	**FSH 5**	**FPH 5**	**FSH 6**	**FPH 6**	**MSH 7**	**MPH 7**
Total number of OTUs	28	18	31	36	19	45	21	32	25	32	20	39	27	36
Temporally stable OTUs	0	3	4	8	1	9	0	8	1	6	0	3	3	2
%Temporally stable OTUs	0	17	13	22	5	20	0	25	4	19	0	8	11	6

Bacterial taxa present in scalp and pubic hairs sampled at three time points over a period of 5 months.

Although temporal stability of an individual’s bacterial taxa may appear to be an important prerequisite for metagenomics to have forensic value, the most relevant attributes will mostly likely be transference of bacteria (during contact), persistence of bacteria post transfer and storage conditions. Consider a case of unlawful sexual intercourse (of an adult female), the most relevant microbial data will be the taxa available for transfer *at the time* of the assault (rather than what it was weeks, months or days before or after) and the persistence of the victim’s bacteria on the offender’s genitals/pubic area (and vice versa). This, of course, is reliant upon collection of evidence from the victim and suspect(s) within several hours of the time of the assault rather than several days. Microbial data from the cohabiting couple, albeit preliminary, are encouraging, in supporting the suggestion of bacterial transfer and persistence following sexual intercourse.

## Conclusions

Despite the modest sample size, we believe that the data in this qualitative assessment of metagenomic analyses of hairs are sufficient to warrant further development of this approach. For this approach to gain traction, there is a need to refine molecular targets—the broad-brush approach of the 16S V4 region looked at here is a good starting point. Additional analyses may provide further information in relation to the microbial composition of ‘core’ microbiomes and their potential value in forensic investigations. However, there is ultimately a need to develop a more focused approach that targets, for example, population level differences within *Lactobacillus* spp. or even more variable genomic sections of common commensals that might contain probative information at a population level.

It is suggested that microbial data gathered from hairs may provide independent data to augment other forensic results, such as mtDNA or YSTR (when DNA yields are sufficient), and possibly provide association between victims of sexual assault and offender, which is currently not possible in the absence of hairs, fibres or seminal fluid. Importantly, conducting metagenomic analyses on hairs does not preclude conducting traditional molecular analyses on the DNA extract.

Despite the complexity of microbial forensic investigations, a substrate such as hair is arguably much simpler to profile than soil a gramme of which may contain up to 50,000 different microbial species [44] or skin, which exhibits high taxonomic divergence and numbers distributed across multiple niches [45]; on the basis of our qualitative assessment, hairs harbour more modest numbers of bacterial diversity. In comparison to scalp hair, pubic hair is somewhat insulated from the environment being colonised with niche specific bacteria. With perseverance, metagenomic analyses of hairs might develop into a useful component of the forensic toolkit to augment existing forensic techniques.

## Abbreviations

BLAST: basic local assignment search tool
BLASTn: Programs search nucleotide databases using a nucleotide query
bp: base pair
emPCR: emulsion-based clonal amplification
FSH: female scalp hair
FPH: female pubic hair
GIT: gastrointestinal tract
MSH: male scalp hair
MPH: male pubic hair
MID: multiplex identifier or ‘barcode’
NGS: next-generation sequencing
qPCR: quantitative polymerase chain reaction
SI: sexual intercourse
